# An Internet-Based and Mobile Family Management Intervention for Mothers of Very Preterm Infants Hospitalized in the Neonatal Intensive Care Unit (the Preemie Progress Program): Pilot Randomized Controlled Trial

**DOI:** 10.2196/66073

**Published:** 2025-05-21

**Authors:** Ashley Weber, Tamilyn Bakas, Qutaibah Oudat, Nehal A Parikh, Joshua Lambert, Heather L Tubbs-Cooley, Jared Rice, Kristin Voos, Matthew Rota, Heather C Kaplan

**Affiliations:** 1 Department of Population Health College of Nursing University of Cincinnati Cincinnati, OH United States; 2 Neurodevelopmental Disorders Prevention Center Perinatal Institute Cincinnati Children's Hospital Medical Center Cincinnati, OH United States; 3 Department of Pediatrics College of Medicine University of Cincinnati Cincinnati, OH United States; 4 Pitzer Center for Women, Children, and Youth College of Nursing The Ohio State University Columbus, OH United States; 5 Department of Pediatrics School of Medicine Case Western Reserve University Cleveland, OH United States; 6 Office of Learning Design and Digital Innovation College of Nursing University of Cincinnati Cincinnati, OH United States; 7 Perinatal Institute Cincinnati Children's Hospital Medical Center Cincinnati, OH United States; 8 James M. Anderson Center for Health Systems Excellence Cincinnati Children's Hospital Medical Center Cincinnati, OH United States

**Keywords:** neonatal intensive care units, preterm infant, family nursing, parents, self-management, family integrated care

## Abstract

**Background:**

Flexible approaches to parenting training interventions in the neonatal intensive care unit (NICU), including family integrated care (FICare) models, are urgently needed across the globe. Many FICare trials inadvertently exclude parents with low resources who cannot commit to daily infant care (eg, 4-8 hours/day). Preemie Progress (PP) is a fully automated, video-based training program that allows parents to choose when and where they learn, without requiring parent bedside presence.

**Objective:**

This study aims to examine the feasibility of recruitment, retention, fidelity, and changes in outcomes during a pilot randomized controlled trial of PP, a video-based intervention aimed at training mothers of very preterm infants in evidence-based family management skills in the NICU.

**Methods:**

Mothers of infants born between 25 weeks and 0 days to 31 weeks and 6 days of gestation were enrolled in an NICU in the Midwestern United States. Electronic surveys were sent to collect maternal outcomes (Patient-Reported Outcomes Measurement Information System [PROMIS] 8a depression and anxiety scales) at baseline (T1), 14 days (T2) and 28 days (T3) after T1, and 30 days after NICU discharge (T4). Infant electronic health records were extracted to collect infant (ie, weight gain velocity at 36 weeks and receipt of mother’s milk) and health care outcomes (ie, NICU length of stay as well as readmissions and emergency department visits within 30 days of discharge).

**Results:**

Of 123 eligible mothers, 64 (52%) were randomly assigned to 1 of 2 arms (PP: n=33, 52%; attention control [AC]: n=31, 48%). Loss to follow-up was 30% (10/33) in the PP arm and 13% (4/31) in the AC arm. PP mothers watched a mean 17.8 (SD 18.9) of 49 videos. PP retention was linked to higher fidelity. PP mothers showed trends toward greater reductions in anxiety 30 days after discharge (mean −7.54, SD 1.93; 95% CI −11.32 to −3.76) compared to AC mothers (mean −4.67, SD 1.59; 95% CI −7.80 to −1.55). PP infants trended toward greater receipt of exclusively mother’s milk 28 days after baseline (PP: 14/26, 54%; AC: 10/28, 36%) and decreased NICU stay (PP: 57.2 days; AC: 68.3 days) but higher readmissions (PP: 4/33, 12%; AC: 2/31, 6%).

**Conclusions:**

We were able to recruit a diverse sample of mothers from a range of socioeconomic backgrounds, including mothers experiencing barriers to bedside presence. Recruitment goals were met. PP showed promising trends in improving maternal, infant, and health care outcomes. Additional studies are needed to optimize PP and study procedures to improve retention and fidelity. PP has the potential to support parent training outside of traditional FICare models or serve as a complement to structure the parent education pillar of adapted FICare models.

**Trial Registration:**

ClinicalTrials.gov NCT04638127; https://www.clinicaltrials.gov/study/NCT04638127

## Introduction

### Background

Increasing numbers of very preterm infants (born at less than 32 weeks of gestation) survive and have chronic, complex health care needs because of prematurity [1]. These infants require long neonatal intensive care unit (NICU) stays and have high health care use, with approximately 50% being readmitted within 90 days of discharge [2-4]. The NICU stay is a unique opportunity for nurses to train families to effectively manage care at the beginning of their infant’s illness trajectory [5]. The duration of hospitalization for these infants is often months, providing parents with extended time to learn about their infant’s care. Nevertheless, parents in the United States and globally typically receive limited structured training to effectively understand, monitor, and manage complex infant care within the chaotic NICU environment [6]. As a result, parents become overwhelmed, disengage early from infant care during NICU hospitalization, and develop depressive and anxious symptoms [7,8].

Flexible approaches to training parents in managing complex infant care in the NICU are urgently needed worldwide. Family integrated care (FICare) is a promising model of care in which parents are integrated into the care team as equal members and are trained by nurses to deliver the majority of infant care in the NICU. FICare has been shown to reduce maternal anxiety and depression [9], improve parenting self-efficacy in the NICU [6], increase infant weight gain and receipt of mother’s milk [10], and decrease NICU length of stay [11]. Various FICare programs have been developed and tested in countries around the world, including Canada, Australia, and New Zealand [10]; China [11]; countries across western Europe [12-14]; and, most recently, the United States [6,15]. Traditional FICare models use an intensive apprenticeship type model, requiring parents to commit to 6 to 8 hours of daily infant care with their NICU nurses, weekly in-person parent education, and presence during medical rounds [14]. In the United States, paid parental leave is not universal, and barriers such as lack of transportation, childcare, and work release make traditional models of FICare untenable for many families. Researchers have recognized that traditional FICare models rely on resource-intensive practices and are designed within specific sociocultural contexts, namely, high-income countries with universal health care and extensive paid parental leave [16]. Thus, adaptations are likely needed for successful implementation in other countries, such as the United States [6]. Very few FICare studies have been conducted in the United States [6,15], and these studies highlight the importance of providing more adaptable approaches toward the FICare pillar of parent education. In sum, adaptations and additional frameworks are needed to structure parent training outside of or within adapted FICare programs to ensure that all parents gain the complex knowledge, skills, and confidence required to care for their infants in the NICU.

### Prior Work

With a clinical academic partnership and deep engagement from key stakeholders [17], we developed an innovative intervention called Preemie Progress (PP) to address the critical need for a feasible parent training program in the United States. PP is a video-based program that teaches evidence-based family management skills informed by the Self- and Family Management Framework (ie, focusing on infant illness needs, activating resources, and living with infant illness) that have been identified by parents of hospitalized preterm infants as being essential in helping them manage care in the NICU [18]. The PP program allows parents to choose when and where they learn, without requiring extensive presence in the NICU. By moving beyond basic information and tasks (eg, diaper changing and feeding) and instead building the parent’s capacity to self-manage the infant’s condition, PP is designed to better prepare parents to execute chronic, complex care in the NICU and at home. Finally, PP was flexibly designed so that this promising intervention could be used as a stand-alone parent training program or as a complement to structure the parent education pillar of adapted FICare programs.

### Goal of the Study

As the initial weeks after birth of a very preterm infant are stressful and chaotic for parents in the NICU, our team needed to understand the feasibility of our intervention and study procedures before a future definitive trial could commence with testing the efficacy of PP.

### Objectives

The aim of our pilot randomized controlled trial (RCT), which has been registered with ClinicalTrials.gov (NCT04638127), was to examine the feasibility of the PP intervention and study procedures within four domains: (1) recruitment (goal: recruit >50% of eligible mothers), (2) retention (goal: achieve <20% loss to follow-up in each trial arm), (3) fidelity to the PP intervention (examine the number and percentage of videos watched by PP mothers), and (4) potential for clinically meaningful changes in our trial outcomes over time (ie, sensitivity to change) throughout the NICU and postdischarge periods.

## Methods

### Design

This pilot study was a prospective, 2-parallel-arm randomized clinical trial with a 1:1 allocation ratio. Mothers of hospitalized preterm infants were randomly assigned to the PP intervention or an attention control (AC) condition within the first 5 weeks of infant life. Mothers, data collectors, and outcome assessors were blinded to trial arm, and data were collected longitudinally across multiple time points. The study was conducted according to the CONSORT (Consolidated Standards of Reporting Trials) guidelines for pilot and feasibility studies (Multimedia Appendix 1) and the TIDieR (Template for Intervention Description and Replication) checklist for interventions (Multimedia Appendix 2 [17-19]). The results are presented according to the CONSORT-EHEALTH (Consolidated Standards of Reporting Trials of Electronic and Mobile Health Applications and Online Telehealth) checklist (Multimedia Appendix 3) [19].

### Conceptual Model

**Figure 1:**
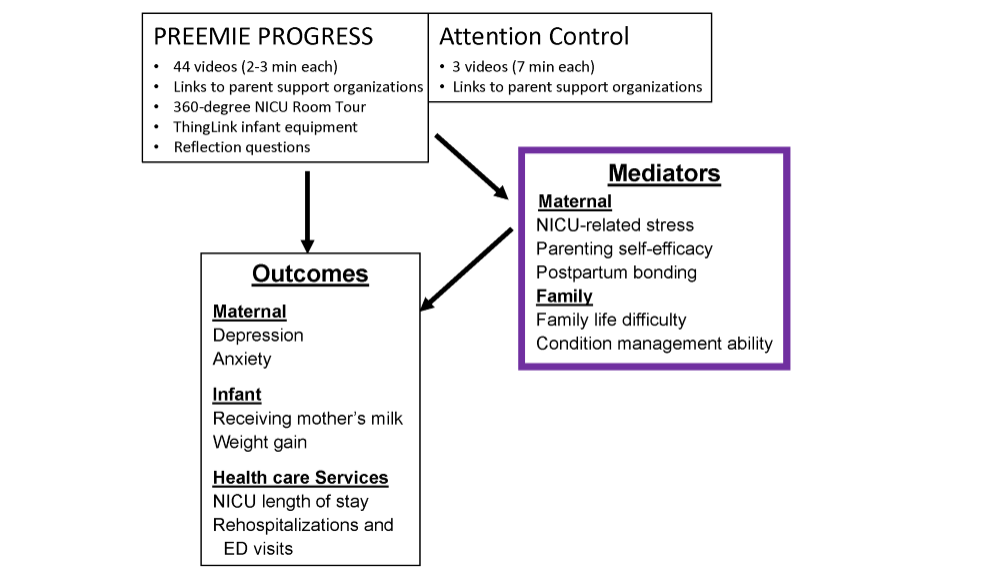
Theoretical model of the Preemie Progress program and its potential impact on outcomes. ED: emergency department; NICU: neonatal intensive care unit.

Figure 1 presents our conceptual model for the study. We believed that the PP intervention would decrease maternal depression and anxiety, increase infant weight gain and receipt of mother’s milk, and decrease neonatal health care use, including NICU length of stay as well as readmissions and emergency department visits within 30 days of discharge. We theorized that this would occur through improvements in maternal parenting self-efficacy, NICU-related stress, postpartum bonding, and maternal ability to manage the infant’s condition and family life difficulties surrounding a NICU hospitalization (Figure 1). As the focus of this pilot trial was feasibility, formal mediation analyses were not conducted because this pilot trial was not powered or intended for such analyses.

### Inclusion and Exclusion Criteria

Mothers were eligible for enrollment if they were English speaking, aged >18 years, and gave birth to infants between 25 weeks and 0 days to 31 weeks and 6 days of gestational age (GA). This GA range provided adequate variability to meet enrollment goals within the study time frame and minimized attrition due to death from extreme prematurity [1]. Due to the pragmatic nature of this study, we included mothers of infants with comorbid conditions (eg, neonatal abstinence syndrome, congenital anomalies, brain injury, and surgical conditions). Mothers were excluded if they did not have custody of their infant, or their infant had imminent or probable risk of death based on the health care team’s judgment.

### Recruitment

Study recruitment and enrollment were conducted during initial infant hospitalization in a level IV NICU in the Midwestern United States. This NICU housed 82 beds and admitted approximately 500 preterm infants per year. All infant rooms are individual or semiprivate family rooms, equipped with free Wi-Fi and unit iPads for education.

Each weekday, study staff screened the NICU electronic health record (EHR) for eligible mothers. Study staff approached eligible mothers during the first 5 weeks of infant life for participation. Mothers were approached either in person or by telephone if in-person contact was not possible after 7 days. After explaining all aspects of the study, study staff sent interested mothers an electronic consent form through REDCap (Research Electronic Data Capture; Vanderbilt University) software.

### Ethical Considerations

Written informed consent was obtained electronically through REDCap from all mothers before participation in this minimal-risk study. This study received ethics approval from the University of Cincinnati Institutional Review Board (2019-0475; Federalwide Assurance: 00003152), and all procedures were conducted in accordance with the Declaration of Helsinki ethical principles and Good Clinical Practice guidelines. Participant privacy and confidentiality were rigorously maintained throughout the study with strict adherence to a data safety and monitoring plan. The principal investigator and her research team held data safety and monitoring meetings quarterly to review protocol deviations, adverse events, study recruitment and retention, data management procedures designed to protect the privacy and confidentiality of participants, relevant literature and conference information, and protocol amendments. All data were deidentified and stored on secure, password-protected servers with access restricted to authorized study personnel. For participation in this study, mothers received “milestone cards” to celebrate steps made toward coming home at the first baseline visit. The cards were valued at approximately US $25 each. Each mother also received a US $25 electronic Amazon gift card for each completed visit after baseline (T1; 14 days [T2] and 28 days [T3] after T1, and 30 days after NICU discharge [T4]) for a total of US $75. No identifiable information is presented in this manuscript or associated supplementary materials.

### Study Interventions and Trial Arms

#### Overview

Mothers were randomly assigned to either the AC arm or the PP arm. Both trial arms viewed their arm’s assigned videos within the trial’s website on their personal devices (ie, mobile phones, computers, or tablets). Both trial arms also received the same number and length of data collection assessments.

#### AC Arm

Mothers allocated to the AC arm received usual care, in which NICU nurses provide verbal education when available at the bedside. To maintain their attention, AC mothers were encouraged to view the welcome videos on the study website that covered topics such as hand hygiene, visitor badges, parking, and the NICU’s unit design.

#### PP Arm

Mothers allocated to the PP arm received usual care and additionally watched PP videos on their personal devices. The PP multimedia content is hosted on the study website and includes 49 videos (each 2-4 minutes in length), review questions, a 360-degree interaction-enabled NICU room tour, and an interaction-enabled ThingLink infant anatomy interactive model that explains equipment commonly used with NICU infants. By accessing short content on their mobile devices, mothers were able to choose when, where, how much, how often, and how quickly they engaged with the material, personalizing the learning experience. The development process for the PP intervention has been previously described [17], and details of the PP program are provided in Multimedia Appendix 4.

### Fidelity to PP and AC Arms

We applied the National Institutes of Health Behavioral Change Consortium’s framework to address the 5 domains of treatment fidelity for both the AC and PP arms [20,21]. Study staff provided a standardized introduction on video access to all parents after baseline visits (training). Mothers within each group viewed standardized video content (design), which was hosted on the university’s secure website portal with advanced analytics capabilities that enabled tracking each mother’s fidelity to their assigned trial arm, defined as the percentage of videos watched over those assigned to the mother (delivery, receipt, and enactment). All mothers received 2 fidelity visits by telephone (approximately 15 minutes each) to answer questions, troubleshoot technology issues, and obtain feedback on the program. We did not set a specific fidelity goal a priori because we wanted to explore the number and percentage of PP videos that were feasible for mothers to watch.

### Instruments With Validity and Reliability

We collected data at T1, T2, T3, and T4. The time points allowed us to assess immediate (T2) and long-term (T3 and T4) sensitivity to changes in outcomes related to intervention implementation. Table 1 lists the outcomes, mediators, measures, and data collection time points. Outcomes were chosen based on demonstrated relationships with family involvement in preterm infant care [22], importance to key stakeholders, and best practices for assessing family involvement with infant care in the NICU [23]. Infant outcomes (ie, weight gain at 36 weeks corrected GA and receipt of mother’s milk) and health care use outcomes (NICU length of stay as well as readmissions and emergency department visits within 30 days of discharge) were collected directly from the infant’s EHR and entered into the trial’s REDCap database.

Maternal outcomes were collected through electronic REDCap surveys sent to mothers at baseline and then 4 days before each postbaseline assessment (ie, T2, T3, and T4) for completion at their leisure. Standardized survey measures were used to assess maternal outcomes, that is, self-reported depressive (Patient-Reported Outcomes Measurement Information System [PROMIS] 8a depression scale) and anxious (PROMIS 8a anxiety scale) symptoms, along with the mediators of condition management ability and family life difficulty (Family Management Measure), NICU-related stress (Parental Stressor Scale: NICU), parenting self-efficacy (Perceived Maternal Parenting Self-Efficacy Scale), and maternal-infant bonding (Postpartum Bonding Questionnaire). These measures have been thoroughly validated, shown to be reliable, and tested with mothers of preterm infants (Table 1).

**Table 1:** Primary outcomes, mediators, measures, and data collection at each study time point in the Preemie Progress trial.

Variables and domains and measures				T1^a^		T2^b^		T3^c^		T4^d^
**Primary outcomes**										
	**Maternal**									
		Depression: PROMIS^e^ 8a T score [24], 8 items; T score standardized to mean 50 (SD 10); higher score indicates greater depressive symptoms; content validity, precision, and reliability established	✓		✓		✓		✓	
		Anxiety: PROMIS 8a T score [24], 8 items; T score standardized to mean 50 (SD 10); higher score indicates greater anxious symptoms	✓		✓		✓		✓	
	**Infant**									
		Receipt of mother’s milk (exclusive, partial, or none)	✓		✓		✓			
		Weight gain at 36 weeks corrected gestational age; *z* score method [10,25,26]					✓			
	**Health care use**									
		NICU^f^ length of stay (days) [27,28]							✓	
		Hospital readmissions and ED^g^ visits within 30 days of discharge [4,29]							✓	
**Mediators**										
	**Maternal**									
		Self-efficacy: Perceived Maternal Parenting Self-Efficacy Scale [30], 20 items (80-point maximum, with higher scores indicating greater self-efficacy); Cronbach α=0.91 and test-retest reliability=0.96; content, convergent, and discriminant validity established	✓		✓		✓			
		NICU-related stress: Parental Stressor Scale: NICU [31], 50 items; 5-point Likert scale ranging from “not at all stressful” (0) to “extremely stressful” (5), with higher scores indicating more stress; Cronbach α=0.87 and test-retest reliability=0.60; content, convergent, and discriminant validity established	✓		✓		✓			
		Maternal-infant bonding: Postpartum Bonding Questionnaire [32], 25 items (125-point maximum, higher scores indicating more impaired bonding); Cronbach α=0.76; content validity established	✓		✓		✓			
	**Family management**									
		Family Management Measure [33] condition management ability scale, 12 items; higher scores indicate greater condition management ability; internal consistency reliability=0.72 and test-retest reliability=0.79; content, convergent, and discriminant validity established	✓		✓		✓		✓	
		Family Management Measure [33] family life difficulty scale, 14 items; higher scores indicate greater family life difficulty; internal consistency reliability=0.90 and test-retest reliability=0.94; content, convergent, and discriminant validity established	✓		✓		✓		✓	

^a^T1: baseline.

^b^T2: 14 days after T1.

^c^T3: 28 days after T1.

^d^T4: 30 days after discharge.

^e^PROMIS: Patient-Reported Outcomes Measurement Information System.

^f^NICU: neonatal intensive care unit.

^g^ED: emergency department.

### Data Collection

After mothers signed the electronic consent form, study staff sent them baseline surveys electronically through REDCap. Mothers without an electronic device were provided with a study tablet to complete surveys. After the baseline surveys were completed, study staff met with mothers in person (or by telephone if unavailable in person) to help them access video content.

### Randomization

Mothers were randomly assigned to 1 of the 2 arms by study staff after baseline data collection to minimize dropout effects on randomization. Using R software (R Foundation for Statistical Computing), the statistician (JL) generated a random allocation sequence stratified by GA at birth (GA <28 weeks and >28 weeks) to balance the representation of the youngest and sickest infants. We used permuted block randomization for each stratum, with block sizes of 2, 4, and 6 and a 1:1 treatment allocation. The statistician (JL) uploaded the randomization sequence into REDCap, had no contact with participants, and was independent of all assessment procedures.

### Blinding

REDCap’s secure randomization feature prevented the study team from viewing the treatment allocation sequence, ensuring allocation concealment. We blinded mothers to trial arm. After randomization, study staff provided mothers with a registration code that would load their assigned video content (ie, AC or PP content) into the study website. Study staff who assessed trial outcomes were blinded through the use of REDCap’s restricted user rights and did not participate in fidelity visits.

### Data Analysis

#### Power

We aimed to enroll at least 60 mothers (n=30, 50% per arm), with planned overrecruitment to account for up to 20% loss to follow-up (ie, 72mothers) during the pilot trial. As we did not have reliable estimates for the CIs, SEs, or mean differences for our outcomes a priori, our sample size was based on the recommendations of Whitehead et al [34], who suggest enrolling at least 20 persons per treatment arm in a pilot trial when expecting a small standardized difference (δ=0.2) to achieve at least 80% power in the main trial. Our recruitment and retention goals, set a priori, were to achieve a recruitment rate of at least 50% among eligible mothers and a loss to follow-up rate of <20% for each arm. As our emphasis was on feasibility, this pilot trial was not powered to detect statistically significant changes in outcome or mediating variables between arms.

#### Analysis Goals and Plan

The primary emphasis of this pilot RCT was to explore data trends surrounding the feasibility domains of (1) recruitment, (2) retention, (3) fidelity to the PP intervention, and (4) sensitivity to changes in our trial outcomes over time, rather than test for statistical significance. Maternal surveys, EHR data, and website analytic data were entered and stored in REDCap and then exported to Stata 17 (StataCorp LLC) software. Descriptive and summary statistics were calculated for all data. Descriptive and summary statistics were used to assess demographic characteristics within each arm, baseline equivalence between arms, and data surrounding the feasibility domains of recruitment (aim to recruit >50% of eligible mothers), retention (achieve <20% loss to follow-up in each arm), fidelity (number and percentage of PP videos watched), and changes in outcomes over time. To understand the impact and potential mechanisms of retention and attrition in the trial, we conducted a post hoc power and statistical analysis to examine whether mothers and their infants differed on demographic characteristics, outcome measures, and fidelity to trial arm. Spaghetti plots with 95% CIs were also used to explore outcome and mediator trends by trial arm. We computed means and 95% CIs for changes from baseline (from T1 to T2-T4) for maternal-reported outcomes by arm. At each follow-up (T2-T4), we also computed mean differences and 95% CIs between arms to evaluate trends in outcomes. To explore trends in NICU length of stay between arms, we computed the mean number of hospitalized days, performed survival analyses, and plotted Kaplan-Meier survival curves for each trial arm.

## Results

### Participant Flow and CONSORT Diagram

**Figure 2:**
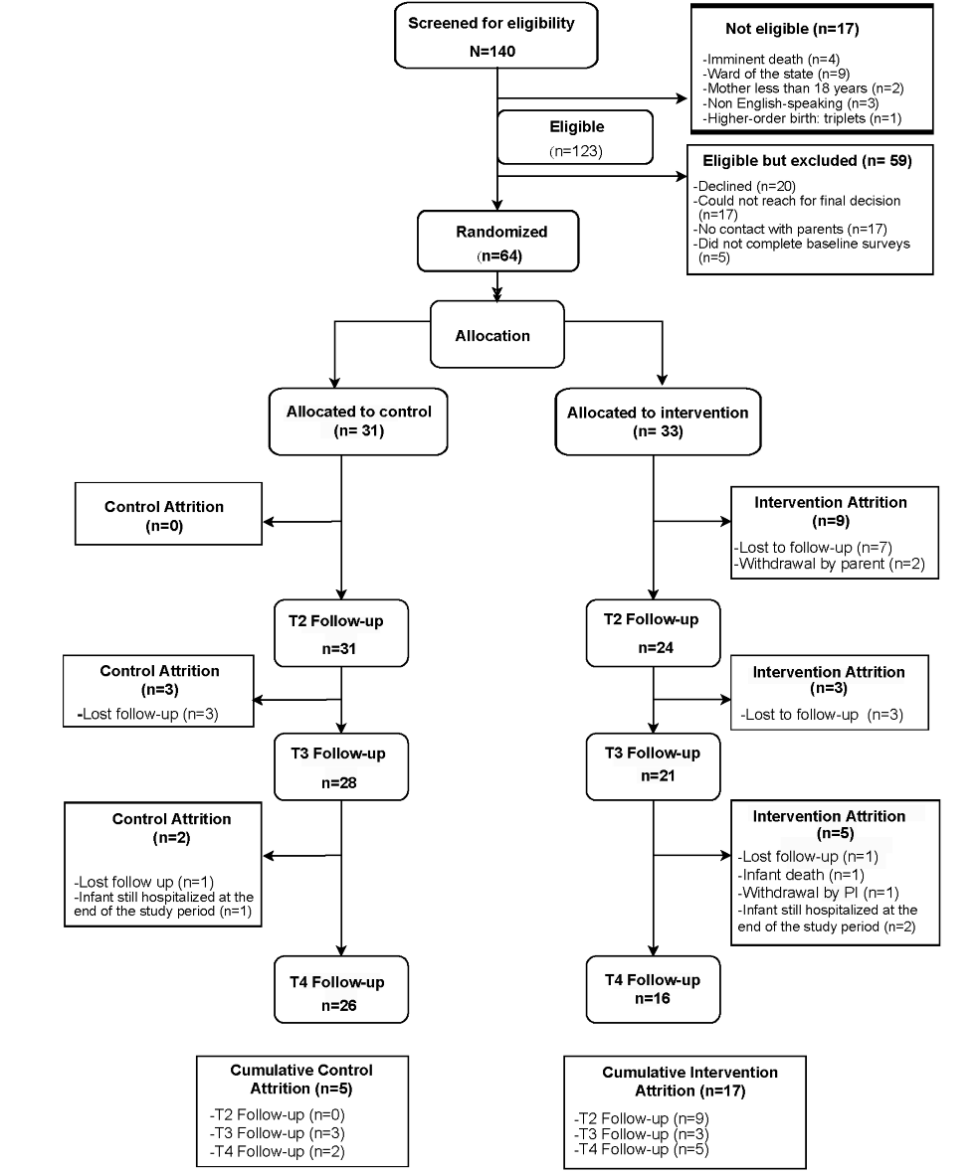
CONSORT (Consolidated Standards of Reporting Trials) diagram for the Preemie Progress pilot randomized controlled trial. PI: principal investigator; T2: 14 days after baseline; T3: 28 days after baseline; T4: 30 days after discharge from the neonatal intensive care unit. Note: mother-infant dyads can be ineligible for more than one reason.

A total of 140 mothers were screened over a period of 14 months (from May 17, 2022, to July 27, 2023), of whom 123 (87.9%) met the eligibility criteria (Figure 2). Of these 123 eligible mothers, 20 (16.3%) declined to participate, with the most frequently cited reasons for declining being not having time and being stressed and overwhelmed. Importantly, study staff were not able to make any contact (eg, telephone call, SMS text message, or in person) with 17 (13.8%) of the 123 eligible mothers, and, after successful initial contact, could not make subsequent contact for a final decision with 17 (13.8%) mothers. These mothers spent limited time in the NICU and did not answer recruitment telephone calls or SMS text messages. Ultimately, of the 123 eligible mothers, 64 (52%) were randomly assigned to the PP arm or the AC arm during the 14-month recruitment period.

Of the 64 mothers, 31 (48%) were allocated to the AC arm. Of these 31 mothers, 4 (13%) were lost to follow-up; and for 1 (3%), the infant was still hospitalized at the end of the study period. Thus, 16% (5/31) of the data for maternal-reported outcomes in the AC arm were missing at T4, and the analyzable AC sample for maternal-reported outcomes at T4 comprised 26 (84%) of the 31 mothers (Figure 2). Of the 33 mothers allocated to the PP arm, 10 (30%) were lost to follow-up; and for 2 (6%), the infants were still hospitalized at the end of the study period; moreover, 3 (9%) withdrew; 1 (3%) was withdrawn by the principal investigator due to severe health issues requiring multiple hospitalizations; and for 1 (3%), the infant died. Thus, “52% (17/33) of the data for maternal-reported outcomes in the PP arm were missing at T4,” and the analyzable PP sample for maternal-reported outcomes at T4 was 16 (48%) of the 33 mothers (Figure 2). Interestingly, mothers who did not complete the study in both arms did not differ on any demographic characteristics or on baseline outcome measures from those who completed the study (Table 2).

**Table 2:** Maternal and infant demographics, baseline outcomes, primary outcomes, and fidelity by study attrition status in the Preemie Progress trial (n=61)^a^.

Variables		Did not complete study (n=19)	Completed study (n=42)	*P* value	Chi-square (*df*) or Mann *U*
**Maternal demographics**					
	Age (y), mean (SD)	27.95 (6.78)	30.52 (5.18)	.17	3.5 (2)
	Education (y), n (%); mean (SD)	17 (89); 13.82 (2.40)	41 (98); 15.24 (2.69)	.16	3.6 (2)
	Income (in US $10,000), n (%); mean (SD)	18 (95); 3.22 (3.98)	41 (98); 5.10 (3.85)	.17	3.6 (2)
**Social determinants of health**		17 (89)	41 (98)		
	No social determinants of health needs, n (%)	10 (59)	32 (78)	.18	3.4 (2)
	Gravida, n (%); mean (SD)	19 (100); 2.79 (1.72)	41 (98); 2.66 (1.85)	.83	0.38 (2)
	Para, mean (SD)	2.00 (1.25)	2.00 (1.23)	.90	0.21 (2)
	Duke Social Support Index score, mean (SD)	18.16 (5.70)	20.60 (5.06)	.20	3.25 (2)
	Marital status: married, n (%)	6 (32)	17 (41)	.82	4.36 (8)
	Race: White, n (%)	8 (42)	26 (62)	.51	9.3 (10)
**Employment status, n (%)**					
	Employed	17 (89)	41 (98)	.85	0.3 (2)
	Not employed in past 12 months	6 (35)	13 (32)	.85	0.3 (2)
**Infant demographics**					
	Gestational age at birth (weeks), mean (SD)	29.56 (1.82)	29.27 (1.94)	.10	4.5 (2)
	Birth weight (g), mean (SD)	1298.84 (398.67)	1292.69 (345.52)	.10	4.7 (2)
	Apgar score (1 min), n (%); mean (SD)	19 (100); 4.74 (2.70)	41 (98); 5.17 (2.62)	.54	1.25 (2)
	Apgar score (5 min), n (%); mean (SD)	19 (100); 6.95 (1.99)	41 (98); 7.76 (1.26)	.24	2.8 (2)
	Day of life at enrollment, mean (SD)	14.37 (9.23)	10.36 (5.91)	.12	4.3 (2)
	Corrected gestational age at enrollment (weeks), mean (SD)	31.61 (1.92)	30.76 (2.03)	.12	1.6 (2)
**Maternal outcomes at baseline**					
	PROMIS^b^ 8a depression scale, mean (SD)	48.64 (8.46)	48.60 (8.23)	.86	0.29 (2)
	PROMIS 8a anxiety scale, mean (SD)	54.12 (7.58)	55.76 (10.58)	.66	0.84 (2)
	Condition (ie, prematurity) management ability, n (%); mean (SD)	18 (95); 47.44 (6.57)	41 (98); 46.61 (6.07)	.85	0.3 (2)
	Family life difficulty, n (%); mean (SD)	18 (95); 27.72 (9.40)	41 (98); 30.95 (11.04)	.73	0.6 (2)
	NICU^c^-related stress: Parental Stressor Scale: NICU, mean (SD)	1.54 (1.04)	1.82 (0.81)	.27	2.6 (2)
	Perceived Maternal Parenting Self-Efficacy Scale, mean (SD)	59.68 (19.91)	52.69 (18.82)	.27	2.59 (2)
	Postpartum Bonding Questionnaire, mean (SD)	4.95 (2.99)	6.02 (7.01)	.76	0.56 (2)
**Infant health care use**					
	NICU length of stay (median time: 50% [days]), median (IQR)	46 (40-80)	49 (44-77)	.44	–0.8
	Hospital readmission or ED^d^ visit within 30 days	4 (21)^e^	2 (5)^e^	*.001^f^*	52.2 (2)
	*z* score change in weight from birth to 36 weeks corrected gestational age, mean (SD)	−0.94 (0.42)	−0.80 (0.69)	.0.3	–1.04
**Receipt of mother’s milk (baseline)**		19 (100)	41 (98)	.48	4.17 (4)
	Infants receiving exclusively mother’s milk, n (%)	8 (42)	24 (59)	.48	4.17 (4)
**Fidelity to PP intervention**					
	Number of videos watched (PP group only), n (%); mean (SD); median (IQR)	15 (79); 4.13 (5.11); 2 (0-8)	17 (40); 28.88 (18.54); 32 (13-49)	*.001*	14.04 (2)

^a^For 3 (5%) of the 64 mothers, their infants were still hospitalized at the end of the study period; thus, they did not have the opportunity to complete or not complete the study. These analyses do not include these mother-infant dyads. Chi-square analyses were used to examine differences between categorical variables (eg, maternal race, marital status, and employment status, as well as infant receipt of exclusive mother’s milk) based on study attrition status. Mann Whitney-*U* nonparametric rank sum tests were used to examine differences between continuous variables based on study attrition status because these variables did not have a normal distribution.

^b^PROMIS: Patient-Reported Outcomes Measurement Information System.

^c^NICU: neonatal intensive care unit.

^d^ED: emergency department.

^e^All 4 mothers who did not complete the study and whose infants were readmitted were in the Preemie Progress (PP) arm of the trial. The 2 mothers who completed the study and had infants who were readmitted were all in the attention control arm of the trial.

^f^Italicization indicates significant *P* values.

### Characteristics of the Sample

Tables 3 and 4 describe baseline demographic and clinical characteristics by trial arm for enrolled mothers and their infants, respectively. As a whole, the mothers (n=64) had a mean age of 29.8 (SD 5.7) years, a mean of 14.8 (SD 2.7) years of education, and a mean income of US $45,400 (SD US $3,900). Nearly 31% (20/64) of the mothers identified at least 1 concern related to the social determinants of health. Of the mothers, 24 (38%) were married. The racial and ethnic composition of our sample reflected the local population, with 36 (56%) of the 64 mothers identifying as White, 23 (36%) identifying as Black or African American, and 62 (97%) identifying as non-Hispanic. Infants were born at a mean of 29.2 (SD 1.9) weeks of gestation with a mean birth weight of 1269 (SD 374) g. Infants were enrolled on average at 11.8 (SD 7.2) days of life, with a corrected GA of 30.9 (2.0) weeks at enrollment. Maternal and infant characteristics were similar between trial arms.

**Table 3:** Demographic, outcome, and mediator measures at baseline for mothers by trial arm in the Preemie Progress trial (n=64)^a^.

Variables				Attention control (n=31)		Preemie Progress (n=33)		*P* value			*t* score (*df*) or Mann *U* or chi-square (*df*)	
**Demographic characteristics**												
	Maternal age (y), mean (SD; range)			30.5 (4.3; 20-41)		29.1 (6.7; 18-46)		.31			1.03 (62)	
	Income (in US $10,000), n (%); mean (SD; range)			30 (97); 44,000 (39,000; 0-100,000)		31 (91); 46,700 (39,000; 0-100,000)		.78			−0.28 (59)	
	**Social determinants of health, n (%)**			31(100)		33(100)		.36			0.83	
		No social determinants of health needs, n (%)	23 (74)		21 (63)		.36			0.83 (1)		
	Years of education, n (%); mean (SD; range)			30 (97); 15 (2.6; 11-19)		30 (91); 14.6 (2.8; 11-21)		.46			0.74	
	Gravida^b^, n (%); mean (SD; range)			31 (100); 2.8 (1.9; 1-7)		32 (97); 2.6 (1.7; 1-9)		.82			0.23	
	Para, mean (SD; range)			2.0 (1.2; 1-5)		2.0 (1.3; 0-6)		.85			−0.18	
	Duke Social Support Index score, mean (SD; range)			20.7 (4.4; 11-30)		19.3 (5.9; 6-30)		.11			1.1 (62)	
	**Marital status, n (%)**								.92			1.55 (4)
		Single, not partnered	9 (29)		9 (27)							
		Married	11 (35)		13 (39)							
		Divorced	0 (0)		1 (3)							
		Partnered, not living together	4 (13)		5 (17)							
		Partnered, living together	7 (23)		5 (17)							
	**Race, n (%)**			30 (97)		33 (100)		.85			3.28 (5)	
		Asian	1 (3)		0 (0)							
		Black or African American	10 (33)		13 (39)							
		White	17 (57)		18 (56)							
		Multiracial	2 (7)		2 (6)							
	**Ethnicity, n (%)**			30 (97)		30 (91)		.48			1.12 (1)	
		Hispanic or Latinx	1 (3)		0 (0)							
		Not Hispanic or Latinx	29 (97)		30 (100)							
	**Employment in past 12 months, n (%)**			30 (97)		30 (91)		.41			1.20 (1)	
		Yes	22 (73)		18 (60)							
		No	8 (27)		12 (40)							
**Baseline maternal outcomes,** **mean (SD; range)**												
	PROMIS^c^ 8a anxiety scale			57.3 (10.4; 37.1-76.9)		53.4 (8.8; 37.1-67.8)		.08			1.76	
	PROMIS 8a depression scale			49.3 (8.0; 38.2-60.3)		48.1 (8.6; 38.2-65.9)		.43			0.79	
**Baseline maternal mediators,** **n (%); mean (SD; range)**												
	Family life difficulty			30 (97); 30.1 (10.3; 15-53)		31 (94); 29.7 (10.9; 15-54)		.95			0.96	
	Condition management ability			30 (97) 46.5 (5.7; 33-60)		31 (94); 47.2 (6.7; 34-60)		.89			−0.15	
	Maternal parenting self-efficacy			31 (100); 51.5 (18.6; 13-80)		33 (100); 58.1 (19.8; 8-80)		.11			−1.61	
	Maternal postpartum bonding			31 (100); 5.3 (5.7; 0-23)		32 (97); 5.9 (6.4; 0-30)		.1			-1.66 (61)	
	NICU^d^-related stress			31 (100); 1.7 (0.8; 0.4-3.3)		33 (100); 1.7 (1.0; 0.2-4.3)		.68			0.42	

^a^None of the maternal demographic characteristics, outcome measures, or mediator measures were statistically different between trial arms at baseline. Mothers were not required to self-report any questions they did not wish to answer. Thus, some data are missing.

^b^Gravida information is missing for 1 mother, whose gravida status was unknown in the electronic health record because she had a series of miscarriages.

^c^PROMIS: Patient-Reported Outcomes Measurement Information System.

^d^NICU: neonatal intensive care unit.

**Table 4:** Demographic characteristics of very preterm infants by trial arm (n=64)^a^.

Variables			Attention control (n=31)		Preemie Progress (n=33)		*t* test (*df*) or Mann *U* or chi-square (*df*)		*P* value
Gestational age at birth (weeks), mean (SD; range)			29.3 (2.1; 25.4-31.9)		29.2 (1.8; 25-31.6)		0.3		.77
Birth weight (g), mean (SD; range)			1286.2 (359.2; 670-2130)		1253.9 (392.4; 480-2050)		0.3 (62)		.73
Apgar^b^ score (1 min), n (%); mean (SD; range)			30 (97); 5.3 (2.5; 1-9)		33 (100); 4.6 (2.8; 0-9)		0.8		.40
Apgar^b^ score (5 min), n (%); mean (SD; range)			30 (97); 7.7 (1.6; 2-9)		33 (100); 7.1 (1.8; 1-9)		1.6		.12
Day of life at enrollment, mean (SD; range)			10.2 (5.6; 2-23)		13.3 (8.1; 1-37)		−1.6		.11
Corrected gestational age at enrollment (weeks), mean (SD; range)			30.7 (2.3; 25.9-35.1)		31.1 (1.9; 26.9-34.2)		−0.8 (62)		.44
Sex: male, n (%)			23 (74)		17 (52)		3.5		.06
**Race, n (%)**							2.2		.75 (3)
	Asian	0 (0)		1 (3)					
	Asian, Black	0 (0)		1 (3)					
	Black	12 (39)		14 (42)					
	White	19 (61)		17 (52)					
**Ethnicity, n (%)**							1.1		.48 (1)
	Not Hispanic or Latinx	1 (3)		0 (0)					
	Hispanic or Latinx	30 (97)		33 (100)					
Infant and mother with positive toxicology screen: no, n (%)			31 (100)		33 (100)		1.0		.99 (1)
**Infant required surgery, n (%)**							1.0		.32 (1)
	No	31 (100)		32 (97)					
	Yes	0 (0)		1 (3)					
**Grade III or IV IVH** ^c^ **or brain injury, n (%)**							0.002		.99 (1)
	No	30 (97)		32 (97)					
	Yes	1 (3)		1 (3)					

^a^None of the infant demographic characteristics were statistically different between arms.

^b^Apgar score for 1 infant was not documented in the electronic health record.

^c^IVH: intraventricular hemorrhage.

### Feasibility Domain: Recruitment and Retention

We met our recruitment goal of recruiting >50% of eligible mothers (64/123, 52%) during our 14-month recruitment period. However, we did not meet our retention goal in the PP arm, which experienced a 30% (10/33) loss to follow-up rate versus the anticipated 20%. This resulted in an analyzable sample size of 16 mothers for PP maternal-reported outcomes at T4. To understand the impact that attrition might have on initial estimates for our outcomes, we conducted a post hoc power analysis using the methods recommended by Whitehead et al [34], discussed earlier. Even when using the smallest (ie, the most conservative) standardized difference between trial arms at any time point for the primary outcomes of anxiety and depression (ie, T3 PROMIS 8a depression scale=0.29), we achieved a standardized difference much higher than expected (δ=1.06 calculated as [(0.29) / (2.18 / sqrt (64)]), indicating that a final sample size of 10 mothers per arm would have been sufficient for this trial and future trials.

### Feasibility Domain: Fidelity to PP Intervention

Mothers in the PP arm showed large variation in their fidelity to watching PP videos. They watched a mean of 17.8 (SD 18.9; range 0-49) of 49 videos, with a median of 9 (IQR 2-39) videos, and they logged into the study website a mean of 6 (SD 7; range 0-30) times, with a median of 4 (IQR 1-11) times. They spent a mean of 95 (SD 104; range 0-310) minutes, with a median of 57 (IQR 5-165) minutes, on the website. PP mothers who did not complete the study watched significantly fewer videos (*P=*.0009; Table 2). PP mothers who completed the trial had higher PP fidelity (median 32, IQR 13-49, of 49 videos watched; Table 2).

### Feasibility Domain: Changes in Outcome Variables by Trial Arm

**Figure 3:**
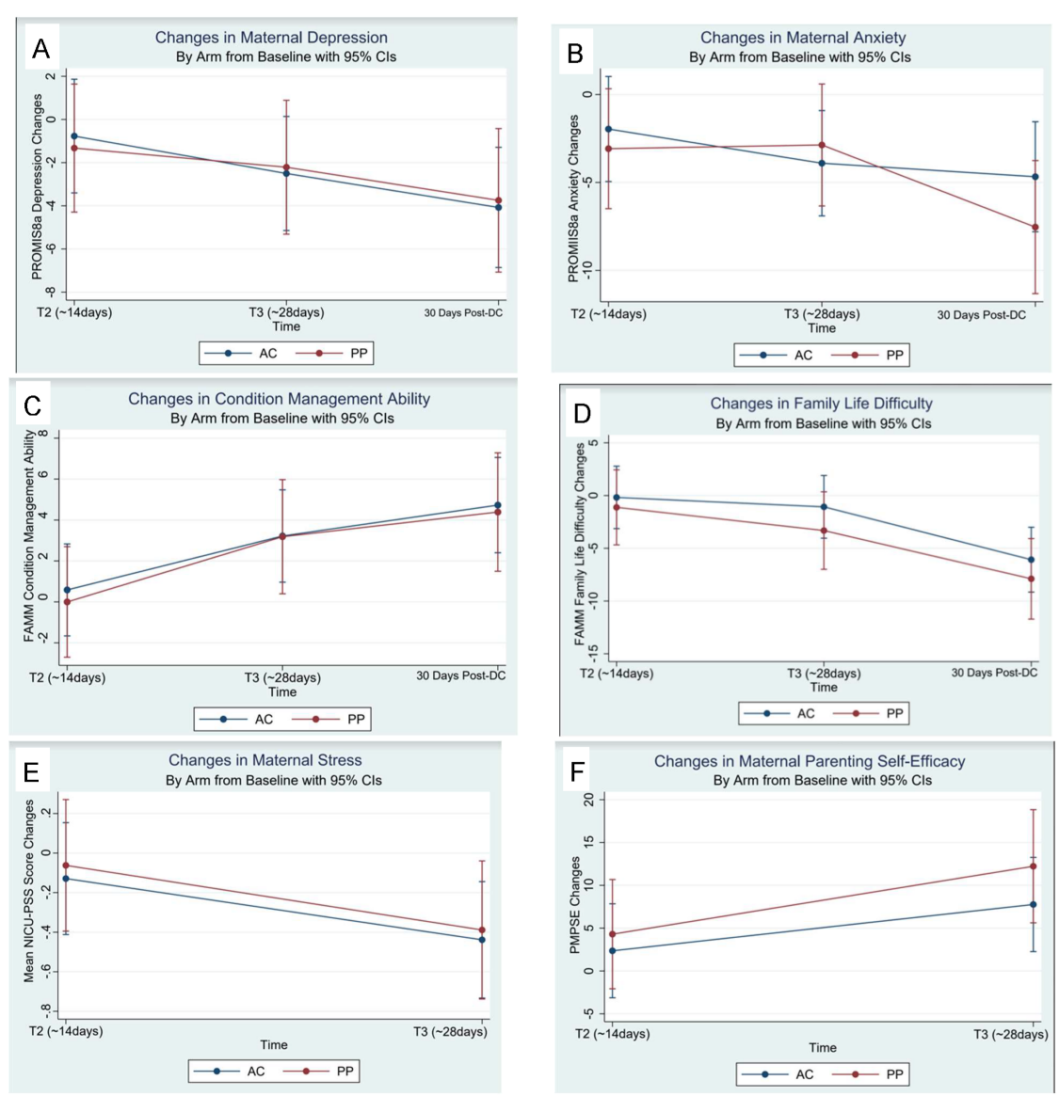
Note: Mothers in the Attention-Control (AC, blue) and Preemie Progress (PP, red) trial arms showed significant decreases from baseline in their PROMIS8a T-scores for depressive (A) and anxious (B) symptoms at 30 days post NICU discharge. Mothers in the PP arm showed a trend towards greater reductions in anxiety (Right). Mothers in both arms reported significant increases in condition (i.e.) management ability at 30 days after hospital discharge (C). Mothers in both arms reported significant decreases in family life difficulty 28 days after baseline and at 30 days post NICU discharge (D). Mothers in both arms reported statistically significant decreases in NICU-related stress (E) and statistically significant increases in parenting self-efficacy (Right) at 28 days post-baseline. Mothers in the PP arm (Red) showed trends towards greater improvements in parenting self-efficacy 28 days after baseline (F).

Descriptive and summary statistics are presented in Table 5 for maternal, infant, and health care use outcomes. PP mothers showed trends toward greater reductions in anxiety (mean −7.54, SD 1.93; 95% CI −11.32 to −3.76) versus AC mothers (mean −4.67, SD 1.59; 95% CI −7.80 to −1.55) 30 days after NICU discharge (Table 6; Figure 3, upper right corner). Both arms showed significant decreases in depressive and anxious symptoms from baseline to 30 days after NICU discharge (Table 6; Figure 3, top row). PP infants showed greater receipt of exclusive mother’s milk (14/26, 54%) versus AC infants (10/28, 36%) 28 days after baseline (Table 5). PP infants showed trends toward decreased NICU length of stay (30/34, 88%; 57.2 days; Table 5) versus AC infants (30/31, 96%; 68.3 days; Table 5). When examined using survival analysis (Figure 3), the median NICU length of stay was similar in both arms, but the PP arm began to show a trend toward decreased length of stay after 51 days (the median). In the face of lower length of stay, the PP arm had higher readmission rates (PP arm: 4/33, 12%; AC arm: 2/31, 6.5%; Table 5). The mothers (4/19, 21%) who did not complete the study and whose infants were readmitted were in the PP arm of the trial (Table 2). Of these 4 mothers, 2 (50%) did not watch any PP videos, and 1 (25%) watched only 2 PP videos. The AC mothers (2/19, 11%) with infants who were readmitted completed the study (Table 2).

**Table 5:** Descriptive and summary statistics for primary outcome measures by trial arm in the Preemie Progress trial (n=64)^a^.

Domains, measures, and time periods			Attention control (n=31)			Preemie Progress (n=33)	
**Health care use outcomes**							
	NICU length of stay (days)^b^, n (%); mean (SD; range)		30 (97); 68.3 (36.3; 26 to 171)			30 (91); 57.2 (23.4; 26 to 114)	
	Hospital readmission or ED visit within 30 days of discharge^c^, n (%)		2 (6)			4 (12)	
**Infant outcomes**							
	*z* score Δ weight (g) from birth to 36 weeks CGA^d^, mean (SD; range); median (IQR)		−0.67 (0.64; −1.6 to 1.8); −0.72 (−1.11 to −0.39)			−0.92 (0.65; −2.6 to 0.95); −0.80 (−1.36 to −0.51)	
**Receipt of mother’s milk^e^, n (%)**							
	**T1**						
		Infants on enteral feeds	30 (97)	33 (100)			
		Infants receiving exclusively mother’s milk	18 (51.5)	17 (48.6)			
	**T2**						
		Infants on enteral feeds	29 (94)		31 (94)		
		Infants receiving exclusively mother’s milk	15 (53.6)		13 (46.4)		
	**T3**						
		Infants on enteral feeds	28 (90)				26 (79)
		Infants receiving exclusively mother’s milk	10 (41.6)				14 (58.3)
**Maternal outcomes, n (%); mean (SD; range)**							
	**PROMIS 8a anxiety scale**						
		T1	31 (100); 57.3 (10.4; 37.1 to 76.9)			33 (100); 53.4 (8.8; 37.1 to 67.8)	
		T2	30 (97); 55.2 (8.8; 37.1 to 69)			25 (76); 51.2 (9.4; 37.1 to 68.8)	
		T3	28 (90); 53.8 (9.1; 37.1 to 68.9)			22 (67); 51.5 (8.5 37.1 to 66.6)	
		T4	25 (81); 52.7 (9.5; 37.1 to 66.9)			16 (48); 47.2 (9.0; 37.1 to 61.5)	
	**PROMIS 8a depression scale**						
		T1	31 (100); 49.3 (8.0; 38.2 to 60.3)			33 (100); 48.1 (8.6; 38.2 to 65.9)	
		T2	30 (97); 47.8 (7.6; 38.2 to 58.5)			26 (79); 46.5 (7.6; 38.2 to 67.6)	
		T3	28 (90); 46.7 (7.9; 38.2 to 62.0)			21 (64); 45.0 (8.3; 38.2 to 63.1)	
		T4	24 (77); 45.6 (7.6; 38.2 to 59.3)			16 (48); 43.3 (6.7; 38.2 to 56.2)	

^a^Health care use outcomes (ie, neonatal intensive care unit [NICU] length of stay as well as readmissions and emergency department [ED] visits within 30 days of discharge) and infant outcomes (ie, weight gain velocity at 36 w and receipt of mother’s milk) were extracted from the infant’s electronic health record 30 days after infant discharge from the NICU. Maternal outcomes (Patient-Reported Outcomes Measurement Information System [PROMIS] 8a depression and anxiety scales) were reported by mothers through REDCap (Research Electronic Data Capture) electronic surveys, which were completed upon enrollment (baseline, T1), 14 days after T1 (T2), 28 days after T1 (T3), and 30 days after infant discharge from the NICU (T4). For continuous variables, we reported the mean, SD, and minimum and maximum. For highly skewed data, we included the median and IQR.

^b^We calculated the mean NICU length of stay in days. We excluded 1 infant in the attention control arm who was still hospitalized at the end of the study period. We excluded 3 infants in the Preemie Progress group: 2 (67%) were still hospitalized at the end of the study period, and 1 (33%) died. Survival analyses that include the censored length of stay for the 4 infants still hospitalized at the end of the study period are presented in Figure 4.

^c^Outcome was coded as “yes” if the infant was rehospitalized or had an ED visit. In this sample, all infants with an ED visit were rehospitalized.

^d^CGA: corrected gestational age.

^e^At each time point, some infants were on intravenous fluids only (ie, nothing by mouth) and were not included in the denominator for this measure.

**Table 6:** Estimated mean changes from baseline (T1) in mother-reported outcomes and mediators by trial arm^a^.

Domains and measures			Attention control (n=31), mean change (SD; 95% CI)		Preemie Progress (n=33), mean change (SD; 95% CI)		Difference in mean change (SD; 95% CI)
**PROMIS^b^ 8a anxiety scale**							
	T2^c^−T1	−1.96 (1.52; −4.95 to 1.02)		−3.08 (1.74; −6.49 to 0.33)		−1.12 (2.47; −5.96 to 3.72)	
	T3^d^−T1	−3.91 (1.53; −6.90 to −0.91)		2.87 (1.77; −6.34 to 0.60)		1.04 (2.46; −3.78 to 5.86)	
	T4^e^−T1	−4.67 (1.59; −7.80 to −1.55)		−7.54 (1.93; −11.32 to −3.76)		−2.87 (2.61; −7.99 to 2.25)	
**PROMIS 8a depression scale**							
	T2−T1	−0.77 (1.34; −3.40 to 1.86)		−1.33 (1.51; −4.29 to 1.64)		−0.56 (2.16; −4.79 to 3.67)	
	T3−T1	−2.50 (1.35; −5.14 to 0.14)		−2.21 (1.58; −5.31 to 0.89)		0.29 (2.18; −3.99 to 4.57)	
	T4−T1	−4.08 (1.42; −6.86 to −1.29)		−3.75 (1.70; −7.07 to −0.42)		0.33 (2.31; −4.19 to 4.85)	
**Condition management ability**							
	T2−T1	0.59 (1.15; −1.66 to 2.83)		0.00 (1.38; −2.70 to 2.70)		−0.59 (1.89; −4.29 to 3.12)	
	T3−T1	3.22 (1.15; 0.96 to 5.47)		3.18 (1.42; 0.40 to 5.97)		−0.04 (1.91; −3.78 to 3.71)	
	T4−T1	4.73 (1.19; 2.40 to 7.06)		4.39 (1.48; 1.50 to 7.28)		−1.54 (1.94; −5.34 to 2.25)	
**Family life difficulty**							
	T2−T1	−0.18 (1.51; −3.14 to 2.78)		−1.12 (1.81; −4.68 to 2.43)		−0.94 (2.49; −5.82 to 3.94)	
	T3−T1	−1.07 (1.52; −4.05 to 1.90)		−3.32 (1.87; −7.00 to 0.35)		−2.25 (2.51; −7.18 to 2.68)	
	T4−T1	−6.08 (1.57; −9.15 to −3.01)		−7.90 (1.95; −11.72 to −4.08)		−1.82 (2.60; −6.92 to 3.28)	
**NICU^f^-related stress**							
	T2−T1	−0.13 (0.14; −0.41 to 0.15)		−0.06 (0.17; −0.39 to 0.27)		0.07 (0.24; −0.40 to 0.53)	
	T3−T1	−0.43 (0.15; −0.73 to −0.14)		−0.39 (0.18; −0.74 to −0.04)		0.05 (0.24; −0.42 to 0.53)	
**Parenting self-efficacy**							
	T2−T1	2.36 (2.80; −3.13 to 7.85)		4.30 (3.25; −2.08 to 10.68)		1.94 (4.59; −7.06 to 10.95)	
	T3−T1	7.76 (2.80; 2.27 to 13.26)		12.23 (3.37; 5.62 to 18.84)		4.46 (4.64; −4.63 to 13.56)	
**Postpartum bonding**							
	T2−T1	0.57 (0.94; −1.28 to 2.42)		1.20 (1.09; −0.94 to 3.34)		0.63 (1.54; −2.40 to 3.65)	
	T3−T1	1.07 (0.95; −0.80 to 2.93)		1.56 (1.13; −0.66 to 3.78)		0.49 (1.56; −2.57 to 3.56)	

^a^Estimates were obtained while controlling for treatment dose (ie, the percentage of videos watched by the mothers).

^b^PROMIS: Patient-Reported Outcomes Measurement Information System.

^c^T2: 14 days after T1.

^d^T3: 28 days after T1.

^e^T4: 30 days after discharge.

^f^NICU: neonatal intensive care unit.

**Figure 4:**
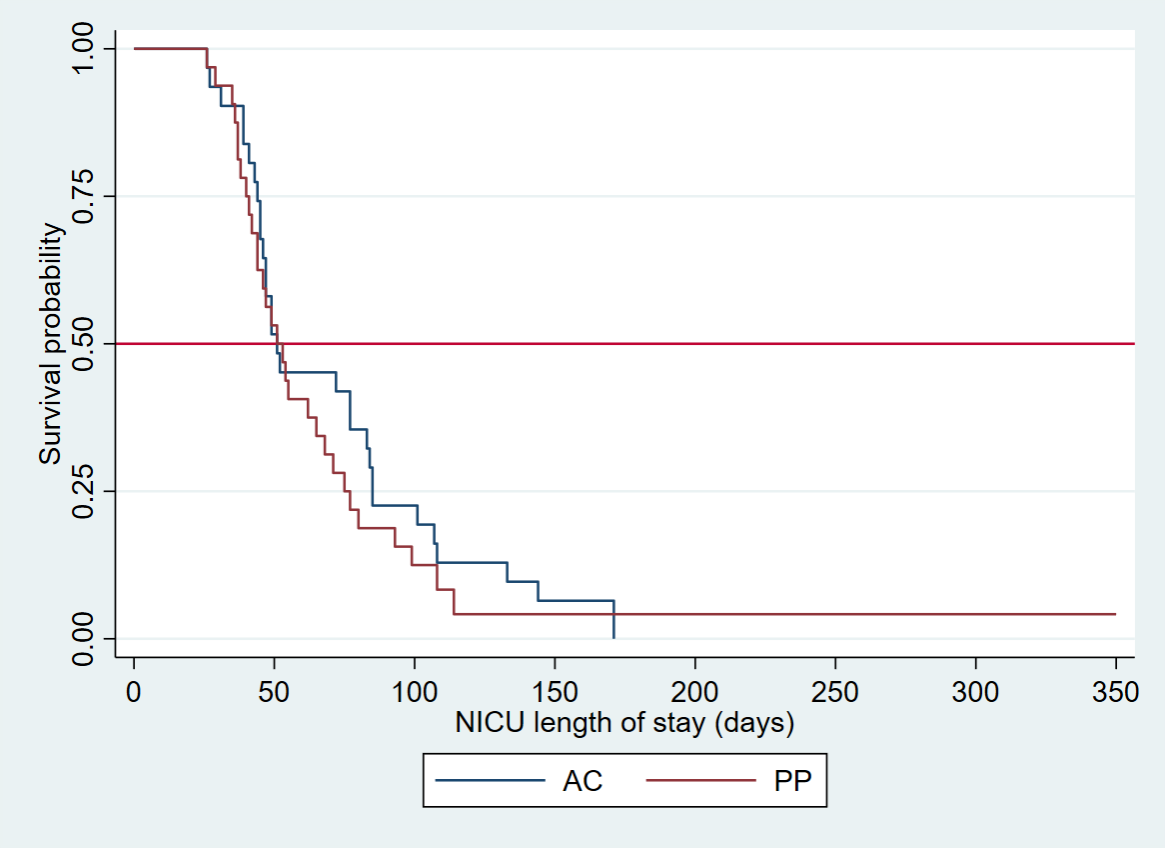
We excluded 1 infant who died in the Preemie Progress (PP) arm. The 4 infants who were still hospitalized at the conclusion of the study were included in this survival analysis and right-censored at the end of the study period. While the median length of neonatal intensive care unit (NICU) stay was similar in both arms (0.5=50%=51 days), the PP arm started to show trends towards decreased length of stay after the 50% median time to NICU discharge. The interquartile lengths of stay at 75% were 44 days for the attention-control (AC) arm and 40 days for the PP arm, and at 25% were 85 days for the AC arm and 75 days for the PP arm.

### Mediator Variables by Trial Arm

Table 7 provides descriptive and summary statistics by arm for our hypothesized mediating variables. Mothers in both arms reported significant increases in condition (ie, prematurity) management ability at T3 and T4 (Table 6; Figure 3, middle left). Mothers in both arms reported significant decreases in family life difficulty at 30 days after NICU discharge (Table 6; Figure 3, middle right). Mothers in both arms reported significant decreases in NICU-related stress and significant increases in parenting self-efficacy at 28 days after baseline (Table 6; Figure 3, bottom row). Mothers in the PP arm showed promising trends for greater improvements in parenting self-efficacy (mean change 12.23, SD 3.37; 95% CI 5.62-18.84) versus AC mothers (mean change 7.76, SD 2.80; 95% CI 2.27, 13.26) 28 days after baseline (Table 6; Figure 3, lower right corner). Mothers in both arms did not have significant or clinically meaningful changes from baseline in postpartum bonding (Table 6).

**Table 7:** Descriptive and summary statistics for mother-reported mediators by trial arm (n=64).

Domains and time periods			Attention control (n=31), n (%); mean (SD; range)		Preemie Progress (n=33), n (%); mean (SD; range)
**Family life difficulty**					
	T1^a^	30 (97); 30.1 (10.3; 15-53)		31 (94); 29.7 (10.9; 15-54)	
	T2^b^	30 (97); 28.7 (10.0; 15-55)		24 (73); 29.1 (11.8; 15-56)	
	T3^c^	28 (90); 28.8 (10.0; 15-54)		19 (58); 26.3 (12.3; 15-55)	
	T4^d^	24 (77); 24.8 (7.3; 15-40)		16 (48); 23.8 (9.6; 15-47)	
**Condition (ie, prematurity) management ability**					
	T1	30 (97); 46.5 (5.7; 33-60)		31 (94); 47.2 (6.7; 34-60)	
	T2	30 (97); 47.3 (6.5; 29-60)		24 (73); 47.8 (6.0; 35-58)	
	T3	28 (90); 50.0 (4.8; 40-60)		19 (58); 51.2 (7.6; 34-60)	
	T4	24 (77); 51.1 (7.1; 39-60)		16 (48); 51.6 (5.8; 39-60)	
**Perceived maternal parenting self-efficacy**					
	T1	31 (100); 51.5 (18.6; 13-80)		33 (100); 58.1 (19.8; 8-80)	
	T2	30 (97); 54.4 (19.2; 0-80)		24 (73); 62.0 (11.9; 33-80)	
	T3	28 (90); 59.0 (19.4; 0-80)		19 (58); 69.6 (10.4; 47-80)	
**Maternal postpartum bonding**					
	T1	31 (100); 5.3 (5.7; 0-23)		33 (100); 5.9 (6.4; 0-30)	
	T2	30 (97); 4.7 (4.3; 0-6)		24 (73); 5.1 (5.1; 0-6)	
	T3	27 (87); 4.1 (4.5; 0-15)		19 (58); 3.9 (5.4; 0-6)	
**NICU^e^-related stress**					
	T1	31 (100); 1.7 (0.8; 0.4-3.3)		33 (100); 1.7 (1.0; 0.2-4.3)	
	T2	30 (97); 1.5 (0.8; 0.0-3.4)		23 (70); 1.6 (1.0; 0-4)	
	T3	26 (84); 1.3 (0.7; 0.3-2.9)		19 (58); 1.3 (0.7; 0.4-2.9)	

^a^T1: baseline.

^b^T2: 14 days after T1.

^c^T3: 28 days after T1.

^d^T4: 30 days after discharge.

^e^NICU: neonatal intensive care unit.

### Adverse Events and Harms

There were no adverse events attributed to this study. One infant died during the study period due to a rare and lethal genetic condition, which was diagnosed after several months of infant life. In our data safety monitoring plan, we anticipated that mothers might experience clinically significant symptoms of anxiety, depression, and psychological distress due to high rates of anxiety and depression in the general population of mothers with infants in the NICU. Three mothers (all in the AC arm) experienced significant self-reported psychological distress or clinically significant symptoms of anxiety or depression (ie, T score >75.0 on the PROMIS 8a anxiety or depression scales), which resulted in the NICU team being notified and referral to mental health support. Symptoms in these mothers improved after referral.

## Discussion

### Overview

This pilot trial is one of the very few studies in the United States [6,15] investigating parent training designed to help parents comanage complex infant care within the first few weeks of a very preterm birth, which can be a traumatic and chaotic time for parents. This discussion is organized around the trial results and recommendations to optimize the feasibility domains of recruitment, retention, fidelity, and sensitivity to changes in outcomes. In addition, the results in this pilot trial are compared to those previously reported by researchers conducting FICare studies in the United States and around the world.

### Feasibility of Recruitment and Retention

It is critical to note that in many FICare trials, parents are either required or highly encouraged to be present at the infant’s bedside 6 to 8 hours a day. As a result, FICare trials may inadvertently include only parents who have the resources to commit to such prolonged, focused time at their infant’s bedside. Unlike our study, in which we could not make contact with approximately 28% (34/123) of the eligible mothers for participation, the FICare study by Franck et al [15] found that approximately 63% of the mothers in their FICare cohort from the United States could not be approached (and therefore recruited) for study participation, and the authors did not distinguish between mothers who could not be approached and those who were ineligible. However, similar to our study, another study by Franck et al [9] reported that 28% and 16% of the enrolled mothers did not complete surveys at infant discharge in their control and mobile FICare cohorts, respectively. The case-control study by Kubicka et al [6] achieved a 90% response rate for maternal surveys, which included instruments used in our study, such as the Parental Stressor Scale: NICU, and the Perceived Maternal Parenting Self-Efficacy Scale; however, the measures were only sent once to mothers at 30 days of infant life; and mothers in the post-FICare cohort were predominantly White (80%), married (96.7%), and highly educated (70% held a bachelor’s degree or higher).

In their FICare trial, van Veenendaal et al [14] found that mothers in the Netherlands who did not complete study surveys were less likely to hold a university degree and be employed and more likely to have infants with longer NICU stays and infants born at less than 32 weeks GA, which are key demographic characteristics of the sample in our study. The high-risk nature of our patient population may explain the higher-than-anticipated loss to follow-up rate in the PP arm. We were surprised to find that mothers who did not complete our study did not differ on any baseline characteristics or on any baseline outcome measures such as family life difficulty and condition management ability. However, mothers in both arms who did not complete the study watched significantly fewer videos. It may be that for these mothers, their life was chaotic in multiple domains, and their ability to complete study procedures and manage their infant’s illness was overwhelming. Very few FICare trials have been conducted in the United States [6,15], and among those conducted, maternal survey measures were typically only assessed at baseline and around discharge [6]. Our design assessed the impact of the PP intervention throughout NICU hospitalization, which may be overwhelming for mothers in the beginning weeks of infant life and may have led to higher loss to follow-up rates.

### Feasibility of Fidelity to PP

In several FICare trials [9,12,14], researchers have found that only mothers who participated in education sessions and medical rounds demonstrated improvements in outcomes such as decreased anxiety. Thus, PP treatment dose and fidelity are likely important for achieving the intended outcomes. A significant strength of our study is that we were able to obtain the number of videos watched for each mother in our study. Mothers in the PP arm watched a median of 9 (IQR 2-39) videos, which suggests that watching all PP videos could be unrealistic for some mothers with infants in the NICU. Similar to our results, Franck et al [15] found in their US trial of a mobile FICare intervention that mothers logged into their mobile education app a median of 10 times during NICU hospitalization, with highly variable maternal use of the We3health app and no effect of the app on infant outcomes. Less than 30% of FICare parents were paired with a parent mentor, and approximately half of the parents attended ≥3 FICare parent group education classes during their infant’s entire NICU hospitalization [15], which we believe further emphasizes the need for parent training interventions that do not rely on in-person methods.

It is imperative that future work around adapted FICare programs optimize the packaging, sequencing, and delivery of web-based parent training. Future trials of the PP intervention could prioritize and segment smaller portions of PP videos to make the intervention feasible. Having mothers select up front what videos they want to focus on may make the PP intervention easier to complete. At the same time, watching only part of the PP program may have implications for PP efficacy. Future trials should also examine how to optimize feasibility with fidelity while preserving the efficacy of the PP intervention.

### Feasibility of Sensitivity to Changes in Outcomes

The results from our pilot trial were consistent with our belief that PP mothers would experience lower anxious symptoms and increased parenting self-efficacy and that their infants would have decreased length of stay in the NICU and increased receipt of mother’s milk. Our trial is also consistent with the systematic review and meta-analysis conducted by North et al [22], which demonstrated that interventions designed to increase family involvement in preterm infant care significantly decrease maternal anxiety, reduce NICU length of stay, and increase infant receipt of mother’s milk. We were surprised that in our sample, PP mothers did not show trends toward greater improvements in maternal depression, NICU-related stress, and postpartum bonding. However, maternal levels of depression, NICU-related stress, and impaired bonding in our sample were considerably lower than those reported by other trials [9,14]. In FICare trials, other researchers have noted that associations between the intervention and maternal mental health symptoms can often only be detected among mothers with initial high burden of mental health symptoms [9]. Thus, additional analyses with subpopulations of mothers with high symptom burden will be warranted in a larger PP trial.

Many of our maternal-reported outcomes only started to improve at 28 days after baseline, which has important implications for future trials of PP. Future trials of PP could consider reducing the number of maternal-reported surveys at 14 days after baseline to decrease study burden because maternal-reported outcome measures did not demonstrate clinically meaningful change at 14 days after baseline. The impact of loss to follow-up on our maternal-reported outcomes could also mean that mothers who were more resilient in multiple areas of life were able to complete survey measures at 28 days after baseline and 30 days after NICU discharge. In many FICare trials, maternal mental health symptoms (eg, stress and anxiety) are only assessed at enrollment and at NICU discharge [12,14,35]. Thus, the impact of FICare on the trajectory of maternal mental health symptoms during NICU hospitalization is not fully known. A significant strength of our study is that our data collection time points at 14 days and 28 days after baseline were standardized for all infants and did not rely on time to infant discharge, which can be highly variable based on infant illness.

### Strengths and Limitations

There are many strengths associated with our pilot trial, including the strong study design; our diverse sample of mothers; a high recruitment rate (64/123, 52%); and the use of blinding, randomization, and validated survey measures. Our use of website analytics allowed us to capture the number of videos watched for each mother, including those mothers who dropped out of the study, which would not have been possible with self-report measures of fidelity. Our use of both in-person and telephone methods of recruitment allowed us to reach a diverse group of mothers, including mothers from a wide range of socioeconomic backgrounds and those who experienced barriers to parent presence in the NICU. Finally, our outcome data showed promising trends for lower anxiety symptoms in the PP arm.

A higher-than-anticipated loss to follow-up rate in the PP arm and large variation in PP fidelity (mean 17.8, SD 18.9 of 49 videos watched) are limitations of this work. The small sample size, while appropriate for a pilot RCT, meant that this study was not powered to detect significant differences in mediator and outcome variables; therefore, the data trends must be interpreted with caution. Our results need to be replicated and confirmed with larger sample sizes.

### Recommendations for Further Research

While we met our recruitment goals, we experienced a higher-than-anticipated loss to follow-up rate in the PP arm. Additional analyses are needed to understand how the PP intervention and study procedures can be refined to optimize PP fidelity and retention rates. Thus, further studies are warranted before a future full-scale efficacy trial can commence. We are currently conducting a companion study analyzing qualitative feedback from mothers in this trial to better understand their experiences with the PP intervention and with contextual factors that influenced their ability to adhere to PP, complete the study, and manage care for their infant. Future studies using mixed methods analyses have the potential to enhance our understanding of the efficacy of PP and offer additional strategies to increase the effectiveness, adoption, and implementation of this intervention for real-world clinical settings.

### Conclusions

Training mothers in evidence-based family management skills has the potential to improve maternal, infant, and health care use outcomes in the NICU and beyond. Mothers in the PP arm showed trends toward greater improvements in anxious symptoms and parenting self-efficacy, and their infants showed trends toward decreased length of stay in the NICU and increased receipt of mother’s milk. While we met our recruitment goals, additional studies are needed to optimize PP and trial procedures so that retention and fidelity goals can be met. However, the PP intervention shows great promise in supporting parent training as a stand-alone program outside of traditional FICare models or as a complement to adapted FICare models in the United States.

## Appendix

Multimedia Appendix 1 CONSORT (Consolidated Standards of Reporting Trials) 2010 checklist.

Multimedia Appendix 2 TIDieR (Template for Intervention Description and Replication) checklist.

Multimedia Appendix 3 CONSORT-EHEALTH (Consolidated Standards of Reporting Trials of Electronic and Mobile Health Applications and Online Telehealth) checklist (V 1.6.1).

Multimedia Appendix 4 Preemie Progress modules.

## Abbreviations

AC: attention control
CONSORT: Consolidated Standards of Reporting Trials
CONSORT-EHEALTH: Consolidated Standards of Reporting Trials of Electronic and Mobile Health Applications and Online Telehealth
EHR: electronic health record
FICare: family integrated care
NICU: neonatal intensive care unit
PP: Preemie Progress
PROMIS: Patient-Reported Outcomes Measurement Information System
RCT: randomized controlled trial
REDCap: Research Electronic Data Capture
TIDieR: Template for Intervention Description and Replication

## Data Availability

The datasets generated and analyzed during this study are not publicly available due to participant confidentiality and ethical restrictions but are available from the corresponding author on reasonable request.
