# Supplementary material for: An Internet-Based and Mobile Family Management Intervention for Mothers of Very Preterm Infants Hospitalized in the Neonatal Intensive Care Unit (the Preemie Progress Program): Pilot Randomized Controlled Trial
Source: JMIR Form Res. 2025 May 21;9:e66073. doi: 10.2196/66073 (PMC12138314; doi:10.2196/66073)
Supplement: Multimedia Appendix 2 [file formative_v9i1e66073_app2.pdf]

See details:

See details:

Supplementary File S2. TIDieR (Template for Intervention Description and Replication) Checklist:

TIDieR
Template for Intervention Description and Replication

Information to include when describing an intervention and the location of the information

Item number	Item	Where located ** Primary paper (page #)	Other † (details)
	**BRIEF NAME**		
**1.**	Provide the name or a phrase that describes the intervention.	1	______
	**WHY**		
**2.**	Describe any rationale, theory, or goal of the elements essential to the intervention.	2-3; Figure 1	______
	**WHAT**		
**3.**	Materials: Describe any physical or informational materials used in the intervention, including those provided to participants or used in intervention delivery or in training of intervention providers. Provide information on where the materials can be accessed (e.g. online appendix, URL).	4	See details: (1–3)
**4.**	Procedures: Describe each of the procedures, activities, and/or processes used in the intervention, including any enabling or support activities.	5-6	______
	**WHO PROVIDED**		
**5.**	For each category of intervention provider (e.g. psychologist, nursing assistant), describe their expertise, background and any specific training given.	1-2; 4	See details: (1-3)
	**HOW**		
**6.**	Describe the modes of delivery (e.g. face-to-face or by some other mechanism, such as internet or telephone) of the intervention and whether it was provided individually or in a group.	4-5	______
	**WHERE**		
**7.**	Describe the type(s) of location(s) where the intervention occurred, including any necessary infrastructure or relevant features.	NA	See details: (1-3)

See details:
(1-3)

	**WHEN and HOW MUCH**		
**8.**	Describe the number of times the intervention was delivered and over what period of time including the number of sessions, their schedule, and their duration, intensity or dose.	4-5	________
	**TAILORING**		
**9.**	If the intervention was planned to be personalised, titrated or adapted, then describe what, why, when, and how.	2; 4	________
	**MODIFICATIONS**		
**10.‡**	If the intervention was modified during the course of the study, describe the changes (what, why, when, and how).	NA	________
	**HOW WELL**		
**11.**	Planned: If intervention adherence or fidelity was assessed, describe how and by whom, and if any strategies were used to maintain or improve fidelity, describe them.	11-12	________
**12.‡**	Actual: If intervention adherence or fidelity was assessed, describe the extent to which the intervention was delivered as planned.	Results	________

** **Authors** - use N/A if an item is not applicable for the intervention being described.

† If the information is not provided in the primary paper, give details of where this information is available:

1.Weber AM, Voos KC, Bakas TM, Rice JB, Blatz MA, Ribeiro APD, et al. A clinical-academic partnership to develop a family management intervention for parents of preterm infants. J Clin Nurs. 2022 Feb;31(3–4):390–405. doi: 10.1111/jocn.15929. PMID: 34219302; PMCID: PMC8724346.

2.Weber AM, Bakas T, Schulman-Green D, Voos KC, Rice JB, Bailey R, et al. Family Management Skills Reported by Parents of Preterm Infants in the NICU using the Self- and Family Management Framework (SFMF). Advances in Neonatal Care, 2(24), 119-131. doi: 10.1097/ANC.0000000000001140. PMID: 38127650; PMCID: PMC10978294.

3.Weber AM. Preemie Progress [Internet]. 2024 [cited 2024 July]. Available from: https://www.preemieprogress.org/

‡ If completing the TIDieR checklist for a protocol, these items are not relevant to the protocol and cannot be described until the study is complete.
