# Supplementary material for: An Internet-Based and Mobile Family Management Intervention for Mothers of Very Preterm Infants Hospitalized in the Neonatal Intensive Care Unit (the Preemie Progress Program): Pilot Randomized Controlled Trial
Source: JMIR Form Res. 2025 May 21;9:e66073. doi: 10.2196/66073 (PMC12138314; doi:10.2196/66073)
Supplement: Multimedia Appendix 4 [file formative_v9i1e66073_app4.docx]

**Supplementary File 3. PREEMIE PROGRESS Modules**

| **Module: Topics** | **Family management processes/tasks/skills** |
| --- | --- |
| **Welcome to the NICU** |  |
| A NICU Intro | Dealing with shock of diagnosis |
| A Preemie’s Story; A Parent’s Story | Processing and sharing emotions; Adjusting to infant illness |
| Each Day is Progress; Make the Most of Your PREEMIE PROGRESS | Meaning making; Integrating infant illness into daily life |
| **Learning in the NICU** |  |
| NICU Room Tour | Acquiring information; controlling environment |
| NICU Baby Equipment | Acquiring information; Learning about regimen |
| How to Learn in the NICU | Modifying lifestyle to adapt to prematurity |
| Work, NICU, Life Balance | Integrating infant illness into daily life  Meaning-making: Re-evaluating life |
| You are your baby’s expert | Psychological resources; self-worth as a parent  Taking ownership of infant health needs; Becoming an expert |
| **You are Important** |  |
| Parenting in the NICU | Psychological resources; advocating for self/family/infant |
| Rounds with your CareTeam;  Medical Rounds | Taking ownership of infant health needs; Becoming an expert  Healthcare resources: navigating the healthcare system |
| **Know your Baby’s Language** |  |
| Intro to Baby’s Language: Touch; Taste; Smell; Hearing; Vision | Taking ownership of infant health needs; recognizing and managing body responses |
| **Your “How-To” Care Kit** |  |
| Diaper, mouth, eyes, ears, nose, and bath care | Taking ownership of infant health needs: Completing health tasks, Becoming an expert: Developing confidence and self-efficacy |
| Kangaroo Care | Health promotion activities; |
| Reducing baby’s stress; Comforting Touch & Massage; Easing Pain | Taking ownership of infant health needs; recognizing and managing body responses |
| **Building Blocks** |  |
| Family Building Blocks | Adjusting; Seeking normalcy in life |
| A Journey of Emotions | Processing and Sharing Emotions |
| Parents and Partners; Mom & Birth Parents; Dads & Partners | Obtaining social support: Modifying lifestyle to prematurity; Seeking normalcy in life |
| **Making and Tracking Progress** |  |
| Tracking Progress; Goal Setting  Make an Action Plan!  Problem Solving in the NICU | Becoming an expert: planning, prioritizing, pacing; goal-setting, decision-making; problem-solving; evaluating effectiveness of family management |
| **Milk is Medicine** |  |
| Cleaning Pump Parts;  Safe Storage & Human Milk | Performing health promotion activities |
| **Progress Pointers** |  |
| Being a NICU mom; Interviews | Obtaining/managing social support |
| **A New Beginning for Progress** |  |
| New Paths to Progress | Meaning-making; personal growth |

*Note:* PREEMIE PROGRESS teaches family management skills informed by the Self and Family Management Framework and have been identified by parents of hospitalized preterm infants as being essential in helping them manage care in the NICU. PREEMIE PROGRESS multimedia content is hosted on a WordPress website, and includes 49 videos (each 2-4 minutes in length), review questions, a 360 NICU Room Tour, and an interactive ThingLink Infant Anatomy virtual model that explains common equipment seen on NICU infants.
